# Open aortic arch repair without circulatory arrest by frozen elephant trunk in Ishimaru zone 0

**DOI:** 10.1186/s13019-024-02671-5

**Published:** 2024-04-13

**Authors:** Ciro Bancone, Alessandro Della Corte, Federica Lo Presti, Rasul Ashurov, Giacomo Sica, Lucrezia Palmieri, Rita Di Fraia, Marisa De Feo

**Affiliations:** 1 Department of Translational Medical Sciences, Unit of Cardiac Surgery, University of Campania “L. Vanvitelli”, V. Monaldi Hospital, Via L. Bianchi, Naples, 80131 Italy; 2 Department of Clinical Internal Medicine, Anaesthesiology and Cardiovascular Sciences, Sapienza University of Rome, Rome, Italy; 3 Radiology Unit, V. Monaldi Hospital, Naples, Italy

**Keywords:** Aortic arch aneurysm, Frozen elephant trunk technique, Circulatory arrest, Hybrid vascular prosthesis, Continuous peripheral perfusion, Auto-regulation

## Abstract

**Background:**

Open arch surgery is technically demanding for the surgeon and surgically and biologically invasive for the patient, requiring a variably long period of hypothermic circulatory arrest.

**Case presentation:**

Here we present a case of an elderly patient with chronic renal failure and multiple splanchnic artery disease successfully treated for a rupturing pseudoaneurysm of the aortic arch with a technique that we developed for particularly frail patients. The procedure includes: triple supra-aortic vessel perfusion; distal thoracic aorta antegrade perfusion; balloon endo-clamping of the descending aorta; and anastomosis of an off-the-shelf hybrid arch prosthesis in Ishimaru zone 0. These maneuvers allowed to maintain an extracorporeal circulation in the phase of distal anastomosis, instead of a period of circulatory arrest, employing just mild hypothermia: technical details are depicted and discussed also in comparison with other methods proposed in the literature.

**Conclusions:**

Being able to take advantage of both open surgery advancements and endovascular methods is the key to cardiovascular surgery success today in front of complex pathologies of the aorta: increasing safety and reducing invasiveness of therapeutic options may progressively extend surgical candidacy to the frailest patients.

## Background

Open surgical aortic arch repair with vascular prostheses implies a circulatory arrest (CA) time in order to perform distal anastomosis: when the disease only involves the aortic arch, distal anastomosis is performed in Ishimaru zone 3, namely beyond the onset of the left subclavian artery. Both CA time and extracorporeal circulation (ECC) time are crucial predictors of early post-operative morbidity and mortality [1]. After the introduction of the frozen elephant trunk (FET) technique with hybrid prostheses, initially intended for treatment of aortic arch diseases also involving the thoracic descending aorta (TDA), proximalization of distal anastomosis has gained more and more popularity. The more easily accessible Ishimaru zone 2 or 1 can be the site of the distal anastomosis, when a previous supra-aortic vessels (SAVs) grafting is accomplished. Even zone 0 anastomosis has been reported with complete de-branching of the arch vessels [2]. In each one of these technical variants, antegrade lower body perfusion is restored as soon as possible, in order to reduce CA time and inherent risks. Aiming at potentially abolishing CA time, different distal antegrade/retrograde perfusion techniques have been described involving use of aortic occlusion balloons placed in the TDA [3]. These strategies may enable aortic arch repair in those patients whose comorbidities and intrinsic frailty make them not eligible for conventional open surgical arch repair, when totally endovascular treatments are not technically feasible [4].

Here we present the case of a frail patient with several comorbidities and an aortic arch pseudoaneurysm successfully treated by implantation of a hybrid prosthesis in Ishimaru zone 0, without CA.

## Case presentation

**Fig. 1:**
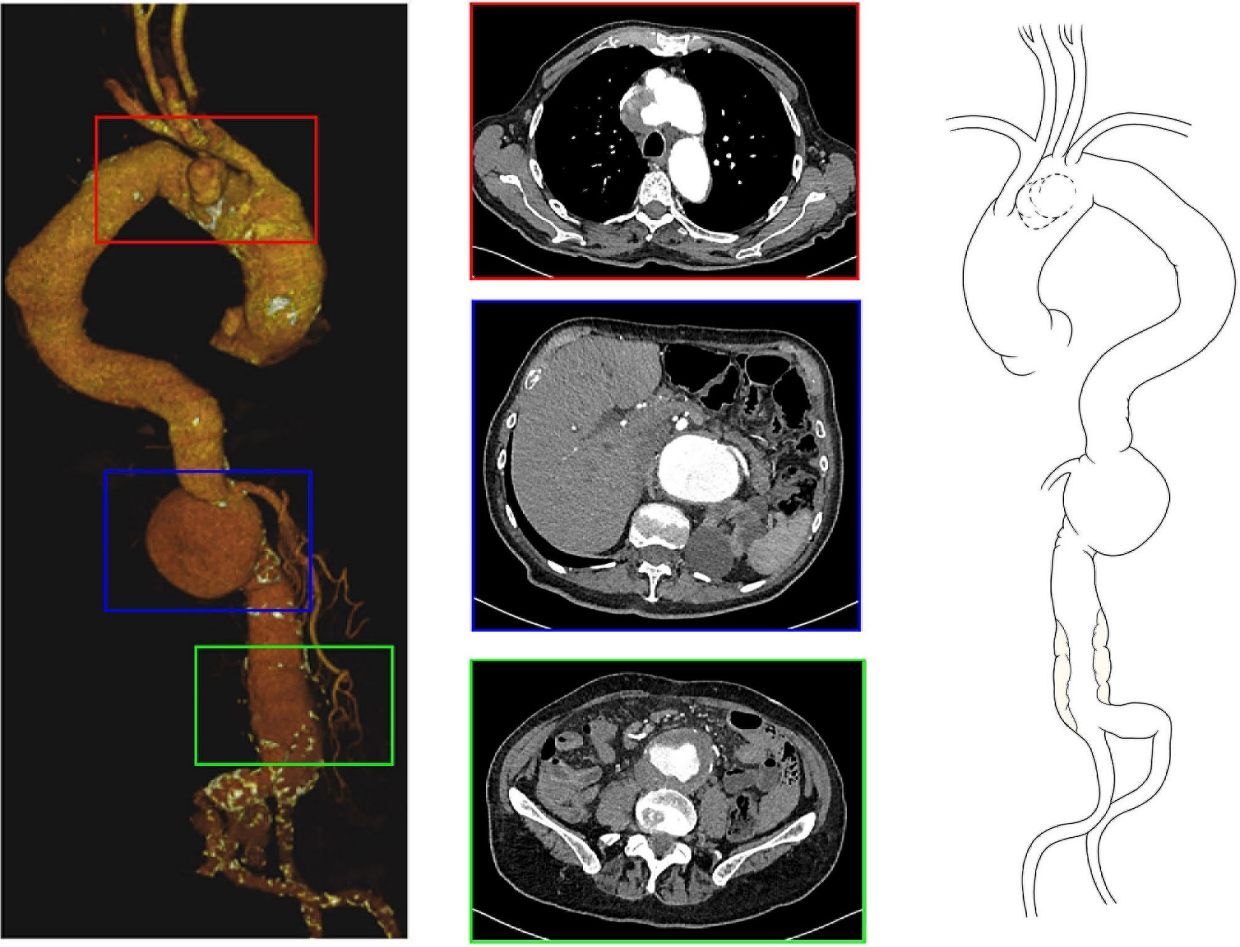
Preoperative CT-scan. On the left, the tridimensional reconstruction (the red box highlights the arch pseudoaneurysm; the blue box the supra-renal abdominal aortic aneurysm; the green box the thrombosed dilated infrarenal segment of the abdominal aorta). In the center, three axial images corresponding to the three pathological segments. On the right, schematic drawing of the patient’s aorta before operation

An 80-year-old male patient suffering from hypertension and stage 3b chronic renal failure was admitted to the emergency department of another hospital for chest pain. His creatinine level was 1.6 md/dL. Total body contrast enhanced computed tomography (CT) showed impending rupture of an aortic arch pseudoaneurysm, originating in zone 2 extending posteriorly and cranially and adhering to the posterior aspect of the brachiocephalic trunk (BCT), in a gothic type III arch configuration. CT scan also showed a 5 cm ascending aortic aneurysm, and a voluminous suprarenal abdominal aortic aneurysm with maximum diameter 8 cm, celiac tripod and inferior mesenteric artery occlusion, renal arteries and superior mesenteric artery patency, and thrombotic apposition of the infrarenal abdominal aorta down to the iliac bifurcation (see Fig. 1).

A less invasive approach like total endovascular arch repair by means of a branched/fenestrated endovascular graft (EVG) or SAV by-passes prior to EVG was not possible since an adequate proximal landing zone was lacking (ascending aortic aneurysm). In such a frail patient, a traditional open surgical arch repair performed with CA and distal anastomosis of the vascular prosthesis in Ishimaru zone 3 would have been burdened by high morbidity and mortality risks, mainly related to the higher splanchnic ischemic risk (being the whole splanchnic district receiving blood only from the superior mesenteric artery) and to a possible worsening of renal failure after CA.

Surgery was performed at the Cardiac Surgery Unit of Monaldi Hospital in Naples, Italy, in a hybrid operative room with a mobile fluoroscopy C-arm. Cerebal perfusion and oxygenation was monitored by means of near infrared spectroscopy (NIRS), whereas continuous arterial pressure monitoring was obtained from catheters into left radial, right radial and left femoral arteries. Median sternotomy allowed for heart, aorta and SAV exposition. Only the left subclavian artery origin was markedly posterior in the aortic arch, hampering intra-thoracic access for its revascularization. Thereafter, by bilateral subclavian incisions, left and right subclavian arteries were isolated; left subclavian offspring was then marked with large hemoclips. A right groin incision was used to access right common femoral artery.

**Fig. 2:**
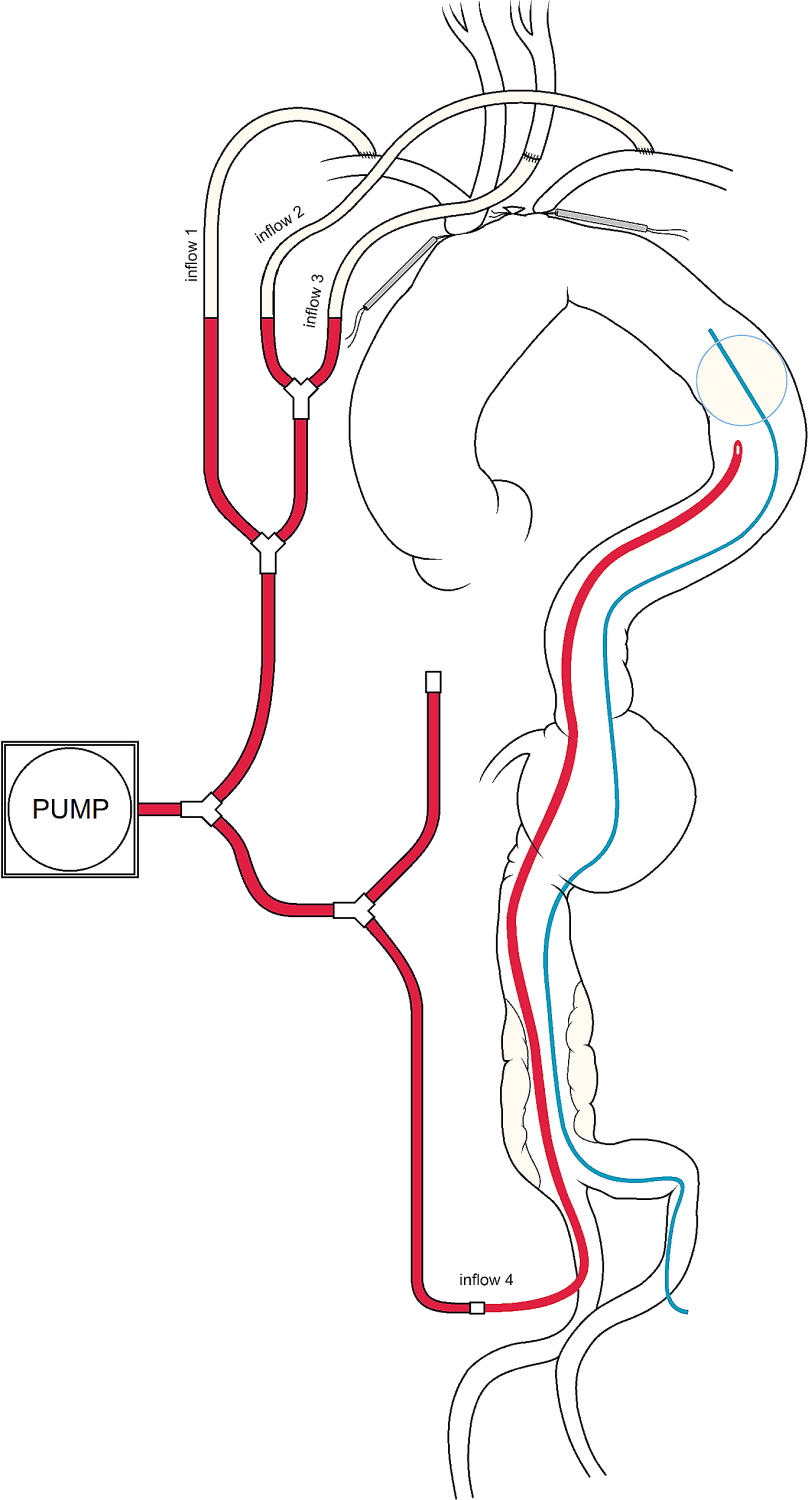
Schematic drawing depicting the customization of a multi-branched arterial line with 5 inflow branches. One was temporarily clamped to be used only after the distal anastomosis (inflow 5); 3 of the other 4 served for perfusion of the SAVs and 1 for TDA antegrade perfusion. Note that with this system, all lines derived from a single centrifugal pump. Two 8-mm prosthetic tubular grafts were anastomosed in termino-lateral fashion to the right and left subclavian arteries and connected to inflows 1 and 2 of the ECC circuit. The left carotid artery was clamped at its origin to check for good left hemisphere NIRS: then it was ligated and transected, then another 8-mm prosthetic tubular graft was anastomosed in termino-terminal fashion to its distal stump and connected to inflow 3. Under fluoroscopic guide, a Reliant balloon (Medtronic, USA) was driven into TDA percutaneously through a 12 Fr introducer sheath from the left femoral artery: a centimeter-marked pigtail catheter allowed for correct positioning of the balloon at 15 cm from the previously marked left subclavian offspring. A long Revas 18 Fr perfusion cannula (Eurosets, Italy) was positioned right below the balloon through the right femoral artery to allow antegrade perfusion in the TDA. This cannula was then connected to inflow 4 (clamped until the balloon was inflated after cardioplegic arrest)

A dedicated ECC circuit was created, using 4 Y connectors and 8 tube segments (all with the same diameter) as shown in Fig. 2: after systemic heparinization, 3 lines were connected to the supra-aortic branches. In the meanwhile, under fluoroscopic guide, a Reliant balloon (Medtronic, USA) was driven into TDA percutaneously, while trans-esophageal echocardiography (TEE) ascertained that the target descending aortic segment was free from dilative/atherosclerotic disease. Just distal to the balloon site, a long Revas 18 Fr perfusion cannula (Eurosets, Italy) was positioned through the right femoral artery to allow antegrade perfusion in the TDA and connected to the fourth branch of the customized circuit (Fig. 2). Antegrade flow in the descending aorta was necessary in this patient to avoid the risk of embolization of clot material from the distal abdominal aorta (where parietal thrombosis was present) to the superior mesenteric artery on which the whole splanchnic perfusion depended. The fifth line was left clamped at this stage of the procedure. The Right atrium was cannulated with a double-stage cannula and connected to the venous line for cardio-pulmonary bypass. ECC was then started, by opening the arterial inflows to the three SAVs (inflows 1, 2 and 3; see Fig. 2). A sump sucker was inserted into the right superior pulmonary vein.

When mild hypothermia (33° C) was achieved, ascending aorta was cross-clamped and Del Nido antegrade cardioplegia administered into the aortic root. Once the heart was arrested, the ascending aorta was unclamped, the BCT and left subclavian artery were clamped at their origin, the TDA endo-clamped by inflating the balloon, and the femoral inflow line (inflow 4, Fig. 2) unclamped: thus, the dilated ascending aorta could be resected and the arch opened in a bloodless operative field, maintaining the patient in ECC and avoiding CA. In this setting, flow distribution was subject to auto-regulation, as for a standard ECC: a deeper hypothermia would have hampered native autoregulatory mechanisms. A Thoraflex hybrid prosthesis (Terumo Aortic, Scotland) 30/40 mm, 100 mm length of the stented portion, with three single branches for SAV re-implantation (12 mm, 10 mm, and 8 mm) and a lateral branch (10 mm) for reperfusion was implanted: the stented portion was released into the aortic arch, thus excluding the pseudoaneurysm, with a distal sealing in zone 3 and the vascular prosthetic collar anastomosed in zone 0 to the aortic arch stump reinforced by Teflon strips. Throughout the period of distal anastomosis, the position of the endoclamp was kept under TEE control (the balloon catheter was fixed outside of the femoral access to avoid dislocation). Once completed the distal anastomosis, systemic perfusion was continued through inflows 1, 2, 3 and 5, clamping inflow 4, deflating the balloon, and removing it, as well as the cannula, from the TDA.

**Fig. 3:**
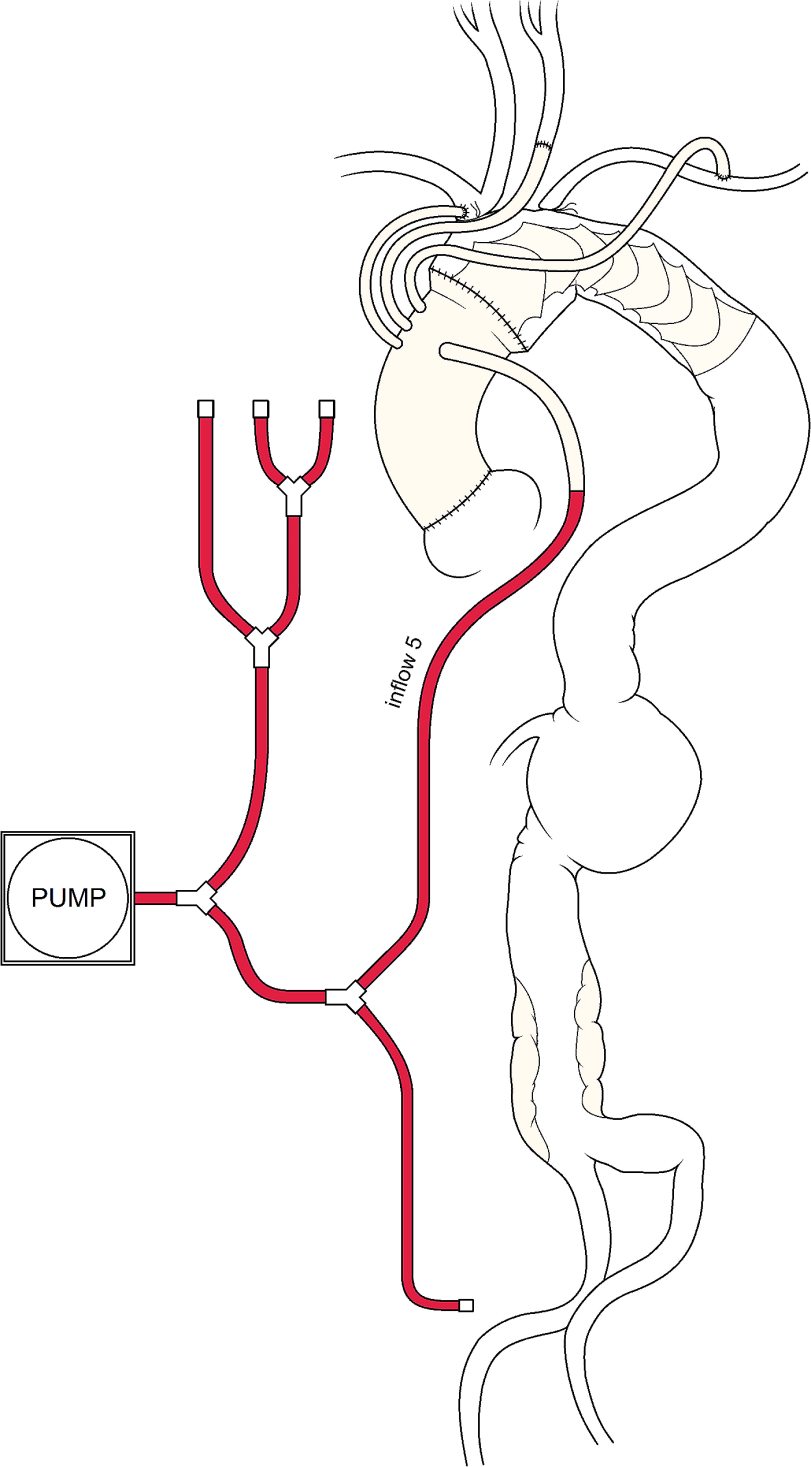
Schematic drawing depicting the completion phase of the operation: with the FET performed in zone 0, the fifth branch of the arterial line was connected to the lateral branch of the prosthesis (inflow 5). During anastomoses of the SAVs, systemic perfusion was continued through lines 1, 2, 3 and 5, after clamping the tubular vascular prosthesis, deflating the balloon, clamping inflow line 4 and removing the femoral cannula. Note that the native origins of all 3 SAVs are ligated. During weaning from CPB, the only line used is inflow 5

**Fig. 4:**
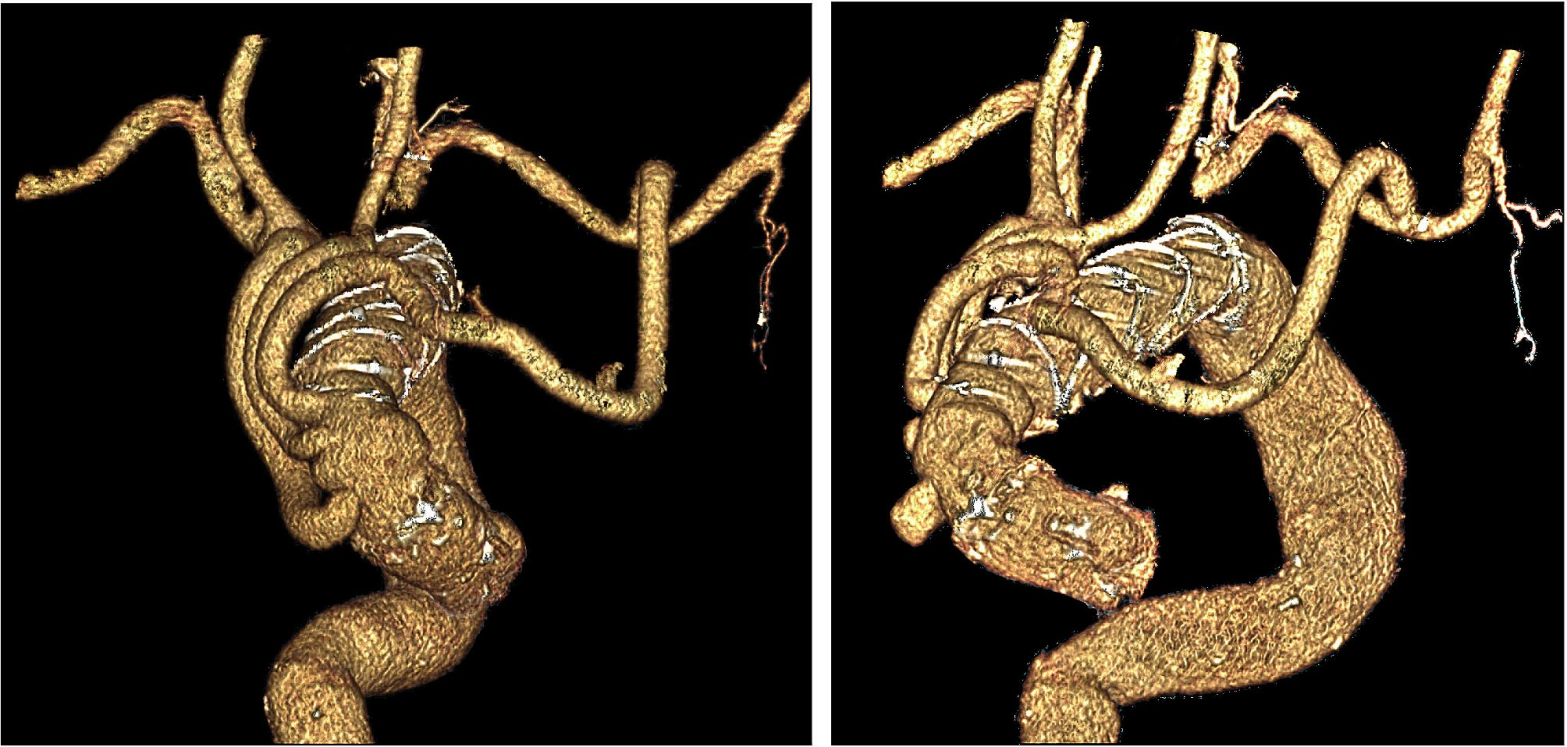
Tridimensional reconstruction of postoperative CT-scan showing complete coverage of the arch and debranching of SAVs from zone 0

The vascular prosthesis was then anastomosed at the sino-tubular junction, de-aired and unclamped. The BCT and the tube graft segment used for left carotid artery perfusion were anastomosed to the first two branches of the prosthesis in a termino-lateral (with side clamping of the BCT) and termino-terminal fashion, respectively (Fig. 3), with the heart beating. ECC was discontinued. The tubular prosthesis previously anastomosed to the left subclavian artery was tunneled into the mediastinum and then anastomosed to the third dedicated branch of the vascular prosthesis (Fig. 3). After protamine administration, BCT and left subclavian artery origins were closed with sutures. Figure 4 shows the final configuration, from 3D reconstruction of postoperative angio-CT-scan. Total ECC time was 203 min, aortic cross clamping time was 84 min. Postoperative course was uneventful and patient was transferred to rehab on postoperative day 18 (creatinine level at discharge from our ward was 1.5 mg/dL).

## Discussion

To the best of our knowledge, this case report represents the first example of aortic arch disease treatment without CA using an off-the-shelf hybrid prosthesis. Others have treated aortic arch diseases without CA, by mean of a stent graft without free flow deployed in zone 2, thereafter used as site of endo-clamping, with subsequent anastomosis of a surgical tubular graft to the stent for replacement of the more proximal aortic segment [3].

In this case, the endo-balloon was advanced from the femoral artery, and retrogradely positioned in the native TDA under both fluoroscopic and TEE guidance. Close monitoring during inflation minimized the risk for aortic injuries. This allowed for a different timing of surgical steps, with aortic endo-clamping preceding the stenting of the aortic arch. Safety and efficacy of endo-aortic balloon occlusion (EABO) has been already previously demonstrated [5]. In different conditions, e.g. with an aortic wall diffusely calcified or atheromatous, a different strategy with descending stenting first should have been chosen.

The advantage of performing a FET operation with a pre-constituted hybrid prosthesis is mainly due to simplification of the distal anastomosis: the collar is directly anastomosed to a teflon-reinforced distal stump of the native aorta, while the more distal segment is already covered. Using two separate prostheses, performing a sort of “reverse elephant trunk”, nullifies the benefits that made FET technique so popular in the last decade [6]. On the other hand, proximalization of the distal anastomosis to zone 0 improves surgical visibility and moves the suture from a higher- to a lower-bleeding-risk site [6], also reducing operative times and minimizing the manipulation of the aortic arch, which is advantageous both for the risk of inferior laryngeal nerve injury and/or, as in the present case, for the risk of rupture of fragile aortic lesions (e.g. pseudoaneurysms) during surgical maneuvers.

Compared to techniques implying zone 2 or 3 anastomosis, our zone 0 anastomosis procedure with a hybrid prosthesis requires methods to ensure flow to the SAVs because their origins are covered. To achieve this, we followed others’ experience of arch surgery with minimization of CA-time [7]: perfusion was provided through a single master centrifugal pump connected to a custom-made multi-line arterial circuit including three lines for SAV direct perfusion and one for the lower body (Fig. 2). All lines had the same diameter: this, together with avoidance of moderate-deep hypothermia, allowed the physiological auto-regulation of blood flow distribution in the different circulatory districts. This is another point of originality of our technical variant compared to other experiences in whom two different pumps were used, necessarily introducing some arbitrariness in choosing empirical flow rates for the two pumps.

Our group’s interest in lower body perfusion during arch surgery dates back to twenty years ago, when FET use had not already spread [8]. As forerunners of the concept of avoiding CA, we employed quite rudimentary means of endo-clamping to perfuse the DTA either antegrade or retrograde, observing beneficial effects in terms of pulmonary and renal complications after moderate-hypothermia arch replacement [8]. Due to the setting, two different pumps were used at that time for SAV and DTA perfusion respectively, in order to maintain control of flow rate in the two different districts since hypothermia interfered with auto-regulatory mechanisms.

Availability of pre-constituted hybrid prostheses has more recently led others to try different strategies for CA-time reduction, depending on distal sealing of the stent graft: if an optimal sealing was the case, either antegrade [9] or retrograde [10] balloon positioning into the stent graft was possible, enabling peripheral retrograde perfusion; otherwise, after stent deployment loops were tightened around the aorta in zone 1 and zone 2 to secure the native aorta around the stent, the surgical graft was clamped and peripheral antegrade perfusion was achieved through its side branch [11].

All the above-mentioned techniques [7–11] needed moderate-deep hypothermia and some CA-time (albeit short), so the inherent risks for systemic untoward effects of CA and the insult to the coagulative system [12] were not avoided: in the present case, hypothermia was mild, not different from our standard valve or proximal aorta operations, and CA-time approached zero. With this regard, it should be underscored that the present case patient was a frail one, importantly affected by chronic kidney insufficiency and diffusely diseased splanchnic arterial circulation.

This technique has recently shown its reproducibility as we have implanted another hybrid prosthesis (Jotec E-Vita Open NEO - Artivion Inc., Atlanta, Georgia) in a patient with aneurysmal ascending aorta and aortic arch with type IA endoleak of a previously implanted EVG in zone 2 for type B aortic dissection. In cases like this the procedure is even facilitated by the fact that a previously implanted EVG ensures effective endo-clamping with the balloon.

## Conclusions and future perspectives

Being able to take advantage of both open surgery advances (use of hybrid prostheses for arch replacement) and endovascular methods and instruments (endo-balloon insertion from the femoral artery) is the key to cardiovascular surgery success today in front of complex pathologies of the aorta: increasing safety and reducing invasiveness of therapeutic options may progressively extend surgical candidacy to more frail patients needing surgical treatments. The field of arch surgery without CA may further advance by implementing new methods: the association of debranch-first techniques, which may help reducing the number of sutures needed to connect the SAV circulation to the aortic prosthesis, thus reducing ECC-time [9]; use of hybrid prostheses specifically manufactured with a configuration favoring zone 0 anastomosis [13]; introduction of new hybrid prostheses with maximum available diameter of the stent graft similar to the EVGs currently available on the market. Since development of chronic aortic disease or acute aortic events proximal to a previously implanted EVG is frequent [14], these prostheses would enable proximal extension of treatment by hybrid prosthesis implantation without CA by our technique.

## Data Availability

No datasets were generated or analysed during the current study.

## Abbreviations

BCT: Brachiocephalic trunk
CA: Circulatory arrest
CT: Computed tomography
ECC: Extracorporeal circulation
EVG: Endovascular graft
FET: Frozen elephant trunk
NIRS: Near infrared spectroscopy
SAV: Supra-aortic vessel
TDA: Thoracic descending aorta
TEE: Transesophageal echocardiography
